# Supplementary material for: Transfer of optical orbital angular momentum to a bound electron
Source: Nat Commun. 2016 Oct 3;7:12998. doi: 10.1038/ncomms12998 (PMC5063962; doi:10.1038/ncomms12998)
Supplement: Supplementary Information — Supplementary Tables 1-2 and Supplementary Notes 1-2. [file ncomms12998-s1.pdf]

Supplementary Tables

**Data Ref.**	**B**	**Beam**	**Pol**	**OAM**	**SAM**	m_{ph}	Δm	Ω_0**(kHz)**	δ **(kHz)**	**P** (μ**W**)	Ω_P**(kHz/**$\sqrt{\mu}$**W)**
**112- 06.07.2015**	+	LG L	L	-1	-1	**-2**	**-2**	13.17(5)	4.70(14)	20.3	**2.92(8)**
105- 06.07.2015	+	LG L	L	-1	-1	-2	-1	3.45(4)	2.52(3)	26.7	0.67(2)
110- 06.07.2015	+	LG L	L	-1	-1	-2	0	0.38(2)	-	25	0.08(1)
108- 06.07.2015	+	LG L	L	-1	-1	-2	+1	1.14(2)	-	21.2	0.25(1)
114- 06.07.2015	+	LG L	L	-1	-1	-2	+2	-	-	17.5	-
86- 08.07.2015	-	Gauss	R	0	-1	-1	-2	3.09(5)	2.2(4)	47	0.45(2)
**75- 08.07.2015**	-	Gauss	R	0	**-1**	**-1**	-1	58.38(16)	14.76(62)	3.5	**31.21(87)**
79- 08.07.2015	-	Gauss	R	0	-1	-1	0	10.82(5)	2.65(19)	55.5	1.45(4)
78- 08.07.2015	-	Gauss	R	0	-1	-1	+1	5.34(4)	1.03(12)	47.5	0.77(3)
83- 08.07.2015	-	Gauss	R	0	-1	-1	+2	-	-	38.5	-
84- 06.07.2015	+	LG L	R	-1	+1	0	-2	0.44(2)	-	20.3	0.10(1)
80- 06.07.2015	+	LG L	R	-1	+1	0	-1	0.80(2)	-	26.7	0.15(1)
**82- 06.07.2015**	+	LG L	R	-1	+1	**0**	**0**	13.92(3)	2.13(19)	25	**2.78(8)**
78- 06.07.2015	+	LG L	R	-1	+1	0	+1	1.70(2)	-	21.2	0.37(1)
86- 06.07.2015	+	LG L	R	-1	+1	0	+2	0.19(2)	-	17.5	0.05(1)
41- 07.07.2015	-	LG L	R	+1	-1	0	-2	0.62(2)	-	21.5	0.13(1)
33- 07.07.2015	-	LG L	R	+1	-1	0	-1	1.75(1)	-	23.9	0.36(1)
37- 07.07.2015	-	LG L	R	+1	-1	**0**	**0**	13.75(1)	0.43(7)	24.5	**2.78(7)**
35- 07.07.2015	-	LG L	R	+1	-1	0	+1	0.62(2)	-	21	0.14(1)
39 - 07.07.2015	-	LG L	R	+1	-1	0	+2	0.23(2)	-	17	0.06(1)
95- 08.07.2015	-	Gauss	L	0	+1	+1	-2	0.27(2)	-	47	0.040 (4)
89- 08.07.2015	-	Gauss	L	0	+1	+1	-1	5.26(5)	2.14(9)	53	0.72(2)
90- 08.07.2015	-	Gauss	L	0	+1	+1	0	3.78(5)	1.98(5)	55.5	0.51(2)
**121- 08.07.2015**	-	Gauss	L	0	+1	**+1**	**+1**	33.84(24)	25.30(32)	3.1	**19.22(62)**
93- 08.07.2015	-	Gauss	L	0	+1	+1	2	4.69(4)	1.46(8)	38.5	0.76(3)
67- 07.07.2015	-	LG L	L	+1	+1	2	-2	-	-	21.5	-
61- 07.07.2015	-	LG L	L	+1	+1	2	-1	1.27(2)	-	23.9	0.26(1)
63- 07.07.2015	-	LG L	L	+1	+1	2	0	0.36(2)	-	24.5	0.07(1)
59- 07.07.2015	-	LG L	L	+1	+1	2	+1	2.81(4)	2.57(3)	21	0.61(2)
**66- 07.07.2015**	-	LG L	L	+1	+1	**+2**	**+2**	5.18(2)	1.46(6)	17	**1.26(4)**

Supplementary Table 1: Fitted Rabi Frequencies Ω_0 and Power normalized Rabi Ω_p for each beam configuration for transitions starting in the $|4^2S_{1/2}, m_J = -\frac{1}{2}\rangle$ state. See Supplementary Note I for details.

**Data Ref.**	**B**	**Beam**	**Pol**	**OAM**	**SAM**	m_{ph}	Δm	Ω_0**(kHz)**	δ **(kHz)**	**P (**μ**W)**	Ω_P**(kHz/**$\sqrt{\mu}$**W)**
**98- 06.07.2015**	+	LG L	L	-1	-1	**-2**	**-2**	5.99(2)	1.11(9)	20.4	**1.33 (4)**
103- 06.07.2015	+	LG L	L	-1	-1	-2	-1	2.80(3)	2.25(3)	22.4	0.59 (2)
97- 06.07.2015	+	LG L	L	-1	-1	-2	0	-	-	24.3	-
94- 06.07.2015	+	LG L	L	-1	-1	-2	+1	1.52(2)	-	24.2	0.31 (1)
100- 06.07.2015	+	LG L	L	-1	-1	-2	+2	-	-	20	-
119- 08.07.2015	-	Gaussian	R	0	-1	-1	-2	2.06(6)	3.35(7)	45.7	0.30(2)
**111- 08.07.2015**	-	Gaussian	R	0	-1	**-1**	**-1**	42.73(11)	7.39(62)	3.2	**23.89(66)**
115- 08.07.2015	-	Gaussian	R	0	-1	-1	0	6.70(3)	1.53(16)	54	0.91(3)
113- 08.07.2015	-	Gaussian	R	0	-1	-1	+1	11.14(1)	0.25(8)	52.5	1.54(4)
117- 08.07.2015	-	Gaussian	R	0	-1	-1	+2	-	-	44.6	-
71 - 06.07.2015	+	LG L	R	-1	+1	0	-2	0.28(1)	-	20.4	0.06(1)
67 - 06.07.2015	+	LG L	R	-1	+1	0	-1	0.50(1)	-	22.4	0.11(1)
**69 - 06.07.2015**	+	LG L	R	-1	+1	**0**	**0**	14.14(3)	2.48(13)	24.3	**2.87(8)**
64 - 06.07.2015	+	LG L	R	-1	+1	0	+1	7.59(5)	4.84(7)	24.2	1.54(5)
73 - 06.07.2015	+	LG L	R	-1	+1	0	+2	0.71(2)	-	20	0.16(1)
28 - 07.07.2015	-	LG L	R	+1	-1	0	-2	-	-	20.5	-
19- 07.07.2015	-	LG L	R	+1	-1	0	-1	1.43(2)	-	22.3	0.30(1)
**24 - 07.07.2015**	-	LG L	R	+1	-1	**0**	**0**	12.91(6)	5.09(14)	24.5	**2.61(8)**
21 - 07.07.2015	-	LG L	R	+1	-1	0	+1	0.80(2)	-	23.5	0.17(1)
26 - 07.07.2015	-	LG L	R	+1	-1	0	+2	0.52(2)	-	19.8	0.12(1)
109- 08.07.2015	-	Gaussian	L	0	+1	+1	-2	-	-	45.7	-
101- 08.07.2015	-	Gaussian	L	0	+1	+1	-1	2.43(5)	2.06(6)	50	0.34(2)
103- 08.07.2015	-	Gaussian	L	0	+1	+1	0	4.15(7)	4.78(6)	54	0.56(2)
**100- 08.07.2015**	-	Gaussian	L	0	+1	**+1**	**+1**	62.84(12)	0.09(5)	3.4	**34.08(92)**
107- 08.07.2015	-	Gaussian	L	0	+1	+1	+2	6.17(3)	0.45(6)	44.6	0.92(3)
55- 07.07.2015	-	LG L	L	+1	+1	+2	-2	-	-	20.5	-
48- 07.07.2015	-	LG L	L	+1	+1	+2	-1	1.03(1)	-	22.3	0.22(1)
51- 07.07.2015	-	LG L	L	+1	+1	+2	0	0.43(2)	-	24.5	0.09(1)
46- 07.07.2015	-	LG L	L	+1	+1	+2	+1	2.53(3)	1.98(4)	23.5	0.52(2)
**53- 07.07.2015**	-	LG L	L	+1	+1	**+2**	**+2**	12.33(4)	3.66(14)	19.8	**2.77(8)**

Supplementary Table 2: Fitted Rabi Frequencies Ω_0 and Power normalized Rabi Ω_p for each beam configuration for transitions starting in the $|4^2S_{1/2}, m_J = +\frac{1}{2}\rangle$ state. See Supplementary Note I for details.

Supplementary Notes

Supplementary Note 1

Supplementary Table I and II show all the measurement settings and results which prove that selection rules depend on the optical orbital angular momentum of photons. The values of Supplementary Table I were used to construct Figure 2b and 2c of the main text. The values of Supplementary Table I and II correspond to analogous experiments where with the initial state are spin -1/2 and +1/2 correspondingly.

The data files for each measurement are also provided as supplementary data as a compressed zip file under the folder *dataRabiFrequencies* and can be referenced by the column Data Ref. of Supplementary Table I.

The total angular momentum of the photon m_{ph} is determined by the direction of the magnetic field B (plus or minus), the polarization (L or R) and type of beam used (Laguerre-Gauss or Gauss). Polarizations R and L are referred to the beam propagation direction. Polarization R is seen by the ion as having spin angular momentum SAM=+1 (σ^+) or SAM=-1 (σ^-) depending on whether the B field is parallel (plus) or anti-parallel (minus) to the direction of propagation of the beam. Correspondingly, the Laguerre-Gauss-left (LG-L) beam is seen by the ion as a beam with orbital angular momentum OAM=-1 or OAM=+1 for corresponding magnetic fields. NOTE: To invert the sense of the OAM, we chose to invert the sense of the magnetic field. From the ion's frame of reference, this is equivalent to changing the sense of rotation of both the Polarization and the OAM of the beam. This was preferred to changing the beam from LG-L to LG-R, which involved realigning the beam on the hologram and the ion. This procedure is very time consuming and less reproducible values as compared to changing the direction of the magnetic field. This fact was confirmed by early measurements.

The difference of angular momentum in a given probed transition $\Delta m = m_f - m_i$ is set by shifting the laser's frequency to be in resonance with the corresponding Zeeman transition.

To estimate the Rabi Frequency per square root micro Watt of power, Rabi Oscillations were measured for each transition and for each beam configuration. The Rabi frequencies Ω_0 were obtained by a non-linear fit to the data of the following function:

$$\frac{\Omega_0}{\sqrt{\Omega_0^2 + \delta^2}} \frac{1}{2}(1 - \cos(2\pi\sqrt{\Omega_0^2 + \delta^2} \cdot t)) \tag{1}$$

A detuning δ is taken into account which arises mainly from stark shifts which lead to an error in the determination of the transition frequency. When less than a quarter oscillation was recorded, we fixed $\delta = 0$ as in this case the fit would otherwise be overdetermined. These were the cases of "off" transitions where we want to give a higher bound to the excitation frequency. The assumption leads to a, possibly, slightly increased fitted Ω_0, which we take as the higher bound. An example of the (time re-scaled) raw data, and the fits can be seen in Figure 2d) in the main text. Non reported values, were all measured to be $\Omega_0 < 0.01kHz$.

The power P for each case was measured before L1 (see Fig. 1 in the main text) with a Thorlabs PM100D+S121C power-meter. The error in the power (not shown but taken into account for the final value) was assumed to be 5%. This was approximately the daily observed overall power fluctuation. We attribute these fluctuations to the laser output power, fibre couplings and diffraction efficiencies of acousto-optic modulators which vary slowly within a couple of hours

The power-normalized values presented in Figure 2d) in the main text are calculated as $\Omega_P = \Omega_0/\sqrt{P}$.

Supplementary Note 2

The raw data and analysis files used to construct Figure 3 of the main text can be found as in the compressed zip provided as supplementary data under the folder *dataProfiles*.
